# Low ceruloplasmin levels exacerbate retinal degeneration in a hereditary hemochromatosis model

**DOI:** 10.1242/dmm.050226

**Published:** 2023-07-13

**Authors:** Brandon D. Anderson, Timothy Lee, Brent Bell, Ying Song, Joshua L. Dunaief

**Affiliations:** FM Kirby Center for Molecular Ophthalmology, Scheie Eye Institute, Department of Ophthalmology, University of Pennsylvania Perelman School of Medicine, Philadelphia, PA 19104, USA

**Keywords:** Ceruloplasmin, Iron, Retina, Hemochromatosis, Oxidative stress

## Abstract

In a previous report, a 39-year-old patient with high serum iron levels from hereditary hemochromatosis (HH) was diagnosed with a form of retinal degeneration called bull's eye maculopathy. This is atypical for patients with HH, so it was theorized that the low serum levels of ferroxidase ceruloplasmin (CP) of this patient coupled with the high iron levels led to the retinal degeneration. CP, by oxidizing iron from its ferrous to ferric form, helps prevent the oxidative damage caused by ferrous iron. To test this, a hepcidin knockout (KO) mouse model of HH was combined with *Cp* KO to test whether the combination would lead to more severe retinal degeneration. Monthly *in vivo* retinal images were acquired and, after 11 months, mice were euthanized for further analyses. Both heterozygous and homozygous *Cp* KO increased the rate and severity of retinal degeneration. These results demonstrate the protective role of CP, which is most likely owing to its ferroxidase activity. The findings suggest that CP levels may influence the severity of retinal degeneration, especially in individuals with high serum iron.

## Teaser

**Summary:** Knocking out the ferroxidase ceruloplasmin in an iron-overloaded mouse model of hereditary hemochromatosis increases the rate and severity of retinal pigment epithelium damage and retinal degeneration.

## INTRODUCTION

Although iron plays an essential role in physiological processes, at high levels it can be toxic to organs sensitive to oxidative stress, such as the eye (Loh et al., 2009). There are several clinical examples of this in the eye. Ocular siderosis occurs when an iron-containing foreign body enters the eye, resulting in rapid photoreceptor and retinal pigment epithelium (RPE) degeneration (Casini et al., 2021). Another example is age-related macular degeneration (AMD), a highly prevalent disease where patients have been found to have higher levels of iron in the retina than age-matched controls (Hahn et al., 2003). Oxidative stress is thought to contribute to AMD pathogenesis (Ruan et al., 2021), and it is hypothesized that oxidative potential of iron can exacerbate the disease, especially when the iron carrier protein transferrin is fully saturated and free iron circulates (Song et al., 2016).

Studies using mouse models have also shown that iron-induced oxidative stress leads to retinal damage and blindness. When iron is administered through intravitreal injection, mouse retinas undergo severe degeneration within a week (Liu et al., 2021; Shu et al., 2020). Conversely, deferiprone, an iron chelator, protects the retina from oxidative stress caused by sodium iodate, an oxidant used to model AMD (Hadziahmetovic et al., 2012). Furthermore, mice that have the iron regulatory hormone hepcidin knocked out experience chronic elevation of serum iron, which leads to retinal damage over the course of a year (Hadziahmetovic et al., 2011).

Another example of apparent retinal iron toxicity is the index case promoting the study described herein. A 39-year-old man was diagnosed with hereditary hemochromatosis (HH), a constellation of genetic diseases characterized by iron overload in multiple organ systems. This case of HH was caused by a homozygous p.C282Y mutation in HFE, which is the most common pathogenic mutation in HH (Feder et al., 1996). He complained of bilateral progressive blurry vision and was diagnosed with bull's eye maculopathy (Bellsmith et al., 2020). This is a degeneration of photoreceptors and RPE cells occurring in a circular pattern within the macula like an archery target bull's eye. Retinopathies have rarely been associated with HH, but in this case it seemed likely because no other genetic or environmental cause of the maculopathy was found and the retinal degeneration did not progress when iron levels were normalized. The patient also exhibited low ceruloplasmin (CP) levels, the underlying mechanism of which was unclear, although it may have been related to low serum copper levels in the patient. It was hypothesized that the combination of low CP and high iron resulted in the bull's eye maculopathy. If so, this would suggest that CP levels play an important role in influencing retinal degeneration.

CP is one of the two extracellular ferroxidases that convert ferrous iron into ferric iron (Osaki et al., 1966). This function is important for protection against iron toxicity for several reasons. First, ferrous iron is more toxic than ferric iron because it generates hydroxyl radicals through the Fenton reaction. These free radicals can then cause oxidative damage to DNA, proteins and lipids, severely reducing their function (Halliwell et al., 2021). Second, iron in its ferric state binds much more readily to transferrin, the main extracellular iron chaperone, which prevents iron from oxidizing other molecules (Dhungana et al., 2004). Third, ferrous iron import into cells is largely unregulated (Sterling et al., 2017), whereas ferric iron import is inhibited by downregulation of the transferrin receptor when intracellular iron levels are high (Rupani and Connell, 2016). The ferroxidase activity of CP thus ensures better iron import regulation. Activity of CP is clearly important for retinal health, as patients with the autosomal recessive disease aceruloplasminemia (no ceruloplasmin) have degenerative changes in the peripheral or macular RPE (Dunaief et al., 2005; Wolkow et al., 2011), in addition to a Parkinson's-like neurodegeneration.

Another iron regulatory protein is hepcidin (HAMP; also known and hereafter referred to as HEPC), which reduces blood iron levels by triggering the degradation of ferroportin (SLC40A1; also known and hereafter referred to as FPN), the only known iron exporter (Nemeth and Ganz, 2021). HEPC can be regulated by homeostatic iron regulator protein (HFE) (Barton et al., 2015). Mutations in *HEPC*, *FPN* or *HFE* can lead to HH, in part by allowing unfettered iron import from the basolateral membrane of gut enterocytes into the bloodstream (Montosi et al., 2001; Roetto et al., 2003). In mouse models of HH, constitutively knocking out *Hfe* and *Hepc* and mutating *Fpn* to be Hepc resistant all resulted in increased retinal iron levels, RPE hypertrophy and autofluorescence, and photoreceptor degeneration (Gnana-Prakasam et al., 2009; Hadziahmetovic et al., 2011; Theurl et al., 2016). In the present study, *Hepc* was knocked out in the liver, its main site of production, using the cre-lox system. The liver-specific knockout (KO) model was used rather than the constitutive KO model because the liver-specific KO model has an increased rate of retinal degeneration. This model causes systemic iron levels to increase as early as 3 months of age and are elevated not only in liver and serum, but also in the retina (Baumann et al., 2019). This retinal iron accumulation can cause oxidative stress, RPE hypertrophy, and subsequent retinal degeneration.

In this study, liver-specific *Hepc* KO (*Cre^+^ Hepc^f/f^*) mice were crossed with *Cp* KO (*Cp^−/−^*) mice to test the hypothesis that low levels of CP would exacerbate the toxicity of high iron levels and cause more severe degeneration, thus modeling the situation in the patient with bull's eye maculopathy.

## RESULTS

### Serum iron levels are elevated by knocking out *Hepc*, and retinal and liver iron are elevated by knocking out *Hepc* or *Cp*

**Fig. 1.**
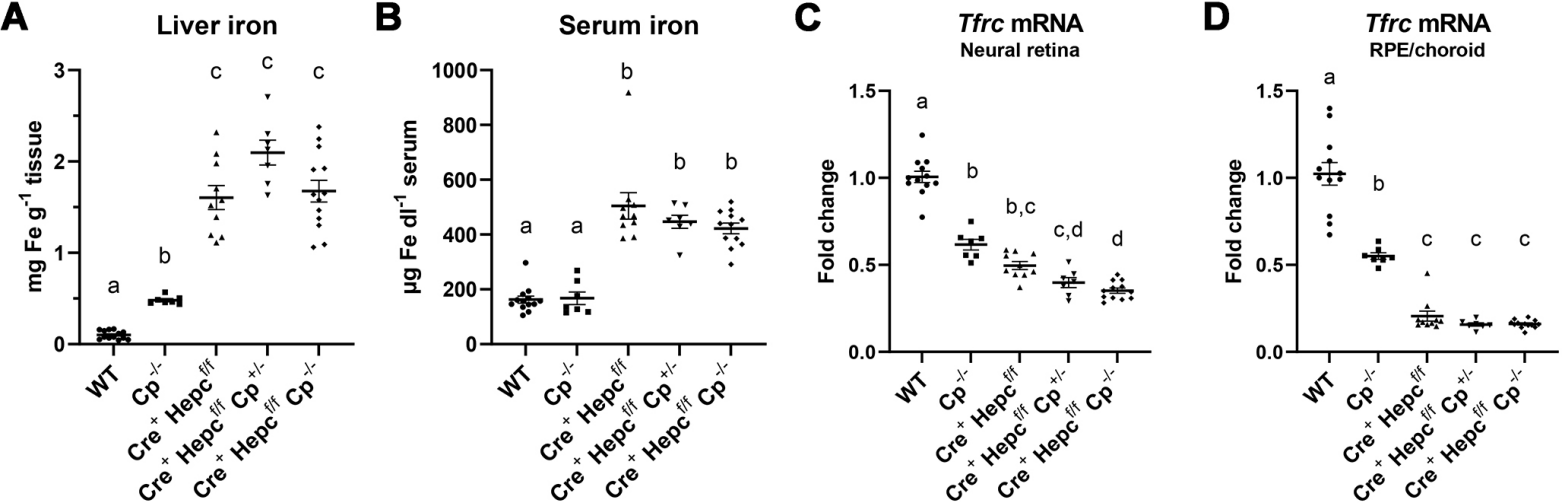
**Increase in iron in liver, serum, and retinal tissue in *Hepc* and *Cp* KO mice.** (A,B) Iron levels in liver (mg iron per g of liver; A) and serum (µg iron per dl serum; B). (C,D) Neural retinal (C) and RPE/choroid (D) *Tfrc* mRNA levels as an indicator of intracellular iron levels; as iron increases, *Tfrc* mRNA is degraded. Bars represent mean±s.e.m. Dots represent individual mice. WT *n*=12; *Cp^−/−^ n*=7; *Cre^+^ Hepc^f/f^ n*=10; *Cre^+^ Hepc^f/f^ Cp^+/−^ n*=7; *Cre^+^ Hepc^f/f^ Cp^−/−^ n*=13 (except B,C *n*=12). Statistical significance was assessed with Brown–Forsythe and Welch ANOVA test followed by Dunnett's T3 multiple comparisons test. Letters above groups designate significant differences: groups that have the same letter are not significantly different from each other.

To confirm that the *Hepc* KO mouse model increased iron levels, 11-month-old mice were euthanized and iron concentrations were measured in the liver, sera, neural retina, and RPE/choroid. Liver iron amounts were increased by an average of 17-fold in all *Cre^+^ Hepc^f/f^* mice, regardless of the presence of *Cp* (Fig. 1A). A milder 5-fold increase was seen in *Cp^−/−^* mice (Fig. 1A). Serum iron levels were only increased in the *Cre^+^ Hepc^f/f^*, *Cre^+^ Hepc^f/f^ Cp^+/−^* and *Cre^+^ Hepc^f/f^ Cp^−/−^* groups, with an average of about three times more iron than wild-type (WT) mice. (Fig. 1B). Intracellular retinal and RPE/choroid iron levels were measured using qRT-PCR analysis of transferrin receptor (*Tfrc*) mRNA (Fig. 1C,D). Because *Tfrc* mRNA contains an iron response element, as intracellular iron levels increase *Tfrc* mRNA is degraded (Rupani and Connell, 2016). By using this method, it was noted that knocking out either *Cp* or *Hepc* caused an increase in iron in the neural retina (Fig. 1C). *Hepc* KO mice had higher levels of iron than *Cp* KO mice, and knocking out both genes caused an even greater increase in iron, regardless of whether one or two copies of *Cp* was knocked out (Fig. 1C). RPE/choroid samples exhibited a dramatic decrease in *Tfrc* in *Hepc* KO samples regardless of the presence of *Cp* (Fig. 1D). The increase in intracellular iron was much greater in the RPE/choroid samples than the neural retina samples (Fig. 1C,D). These iron changes in the liver, serum and retina of the *Cre^+^ Hepc^f/f^* mice are similar to observations in our previous report (Baumann et al., 2019), but the effect of Cp was not studied previously.

### Autofluorescent RPE lesions appear at a younger age in *Cp/Hepc* double-knockout (DKO) mice than in *Hepc* KO mice

**Fig. 2.**
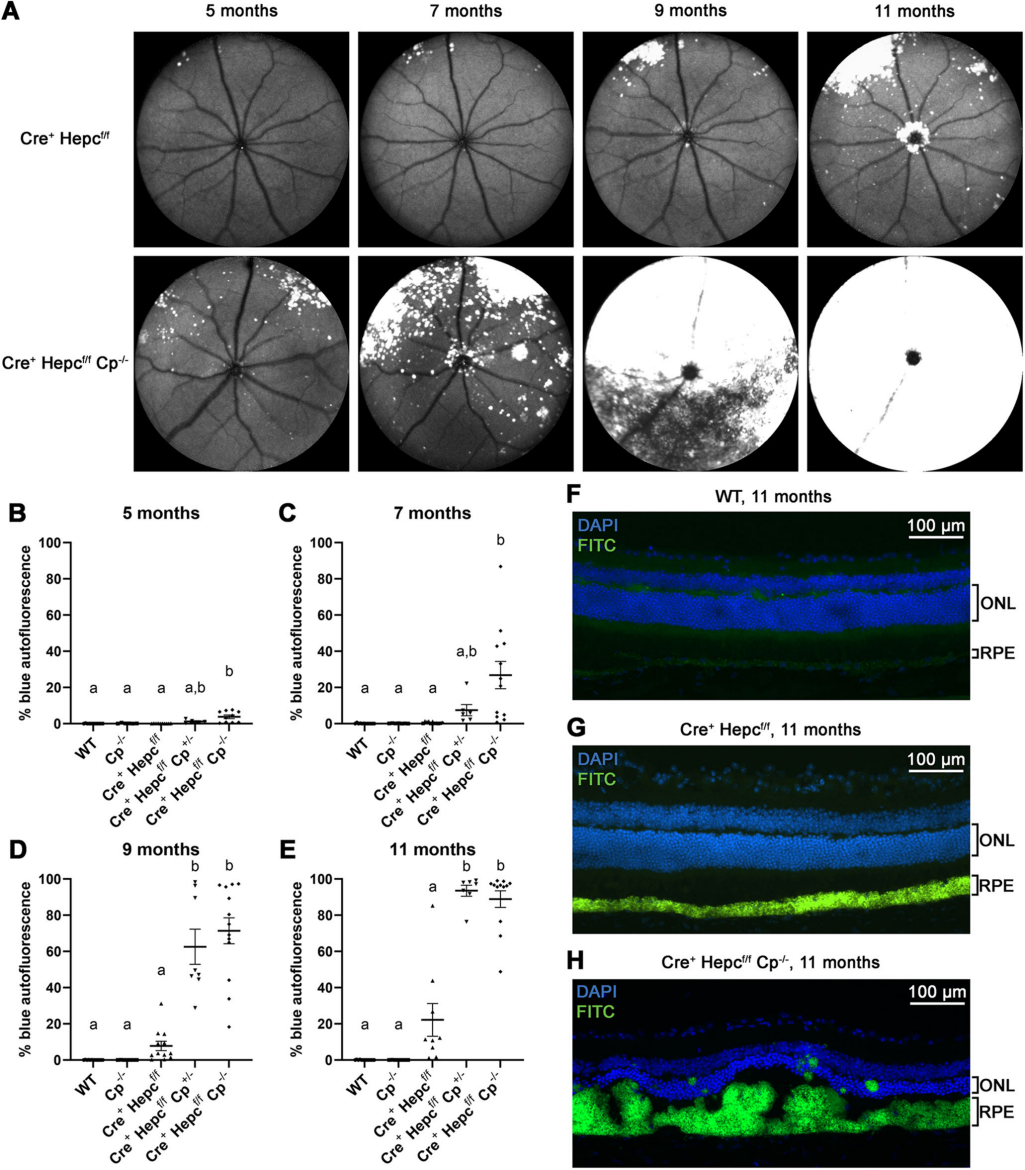
**Occurrence and quantification of blue (488 nm) autofluorescent lesions in the retina.** (A) *In vivo* retinal blue autofluorescent images taken with the cSLO system in one representative *Cre^+^ Hepc^f/f^* mouse and one representative *Cre^+^ Hepc^f/f^ Cp^−/−^* mouse at 5, 7, 9 and 11 months of age. A 105° Ultrawide Field (UWF) lens provides ∼3.4 mm field of view. (B-E) Percentage of image area with blue autofluorescence in each cSLO image (such as those in A) at 5 (B), 7 (C), 9 (D) and 11 months. Mean±s.e.m. Dots represent individual mice (the right and left eyes were averaged together). 5 months: WT *n*=12, *Cp^−/−^ n*=6, *Cre^+^ Hepc^f/f^ n*=10, *Cre^+^ Hepc^f/f^ Cp^+/−^ n*=5, *Cre^+^ Hepc^f/f^ Cp^−/−^ n*=10; 7 months: WT *n*=12, *Cp^−/−^ n*=7, *Cre^+^ Hepc^f/f^ n*=11, *Cre^+^ Hepc^f/f^ Cp^+/−^ n*=6, *Cre^+^ Hepc^f/f^ Cp^−/−^ n*=12; 9 months: WT *n*=13, *Cp^−/−^ n*=7, *Cre^+^ Hepc^f/f^ n*=12, *Cre^+^ Hepc^f/f^ Cp^+/−^ n*=8, *Cre^+^ Hepc^f/f^ Cp^−/−^ n*=13; 11 months: WT *n*=13, *Cp^−/−^ n*=7, *Cre^+^ Hepc^f/f^ n*=9, *Cre^+^ Hepc^f/f^ Cp^+/−^ n*=7, *Cre^+^ Hepc^f/f^ Cp^−/−^ n*=12. Statistical significance was assessed with a Brown–Forsythe and Welch ANOVA test followed by Dunnett's T3 multiple comparisons test. Letters above groups designate significant differences: groups that have the same letter are not significantly different from each other. (F-H) Nuclei (blue) and autofluorescence (green) in fluorescence photomicrographs of DAPI-stained cryosections of WT (F), *Cre^+^ Hepc^f/f^* (G) and *Cre^+^ Hepc^f/f^ Cp^−/−^* (H) mice after they were euthanized at 11 months of age. Note that the RPE was still pigmented as no bleaching protocol was used. Scale bars: 100 µm.

All mouse retinas were imaged once a month starting at approximately 4 months of age and ending when they were euthanized at 11 months. A confocal scanning laser ophthalmoscope (cSLO) was used to monitor the progression of the damage caused by the iron toxicity. Autofluorescent lesions started to appear in the superior retinal region at about 5 months and gradually increased over time (Fig. 2A). The blue (488 nm) autofluorescent lesions were quantified by measuring the percentage of the images containing these lesions (Fig. 2B-E). Although some autofluorescence was seen in the *Cre^+^ Hepc^f/f^* mice as early as 5 months and progressed over time (Fig. 2A-E), the percentage of blue autofluorescent area was never enough to be significantly different from the WT and *Cp^−/−^* groups (Fig. 2E). Conversely, the DKO *Cre^+^ Hepc^f/f^ Cp^−/−^* mice were significantly different from the WT group as early as 5 months of age (Fig. 2B) and eventually the autofluorescence spread pan-retinally by 9 or 11 months (Fig. 2D,E). Interestingly, the *Hepc* KO mice that had one copy of *Cp* (*Cre^+^ Hepc^f/f^ Cp^+/−^*) were phenotypically closer to the *Cre^+^ Hepc^f/f^ Cp^−/−^* group, resulting in no significant difference between the two groups at 9 and 11 months (Fig. 2D,E). After the mice were euthanized at 11 months, the eyes were cryosectioned and the autofluorescence was examined with the FITC filter on an epifluorescence microscope (Fig. 2F-H). All of the autofluorescence seen in the cSLO images (Fig. 2A) originated in the RPE cell layer (Fig. 2G,H). Although both genotypes exhibited autofluorescence, RPE cells in *Cre^+^ Hepc^f/f^* mice were only mildly hypertrophic (Fig. 2G), whereas those in *Cre^+^ Hepc^f/f^ Cp^−/−^* mice had undergone more pronounced hypertrophy (Fig. 2H).

### *Cp/Hepc* DKO mice have RPE hypertrophy and outer nuclear layer thinning

**Fig. 3.**
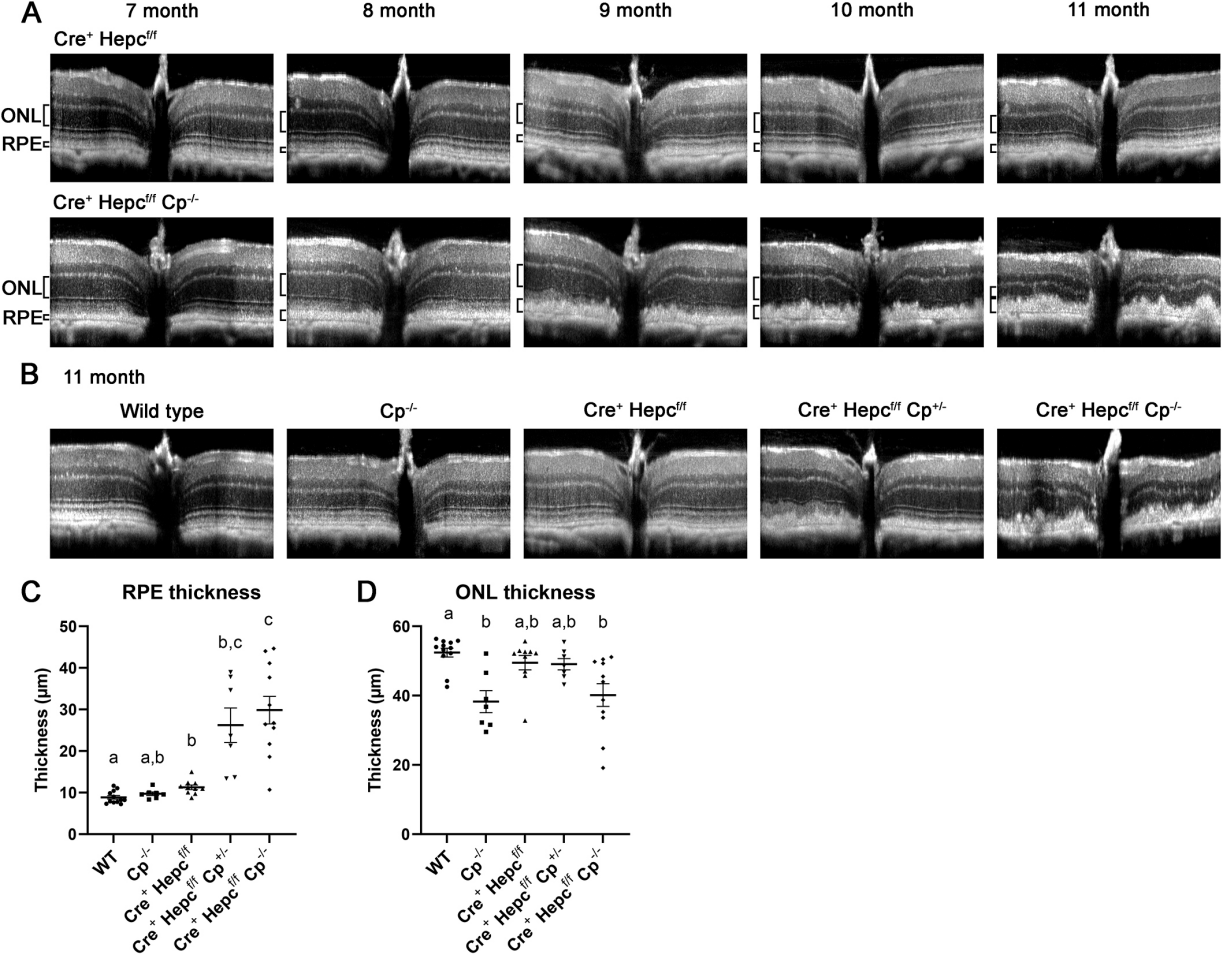
**Thinning of the ONL and progression of RPE hypertrophy over time in *Cre^+^ Hepc^f/f^ Cp^−/−^* mice.** (A) Monthly representative OCT images taken of one *Cre^+^ Hepc^f/f^* mouse and one *Cre^+^ Hepc^f/f^ Cp^−/−^* mouse from 7 to 11 months of age. Photoreceptor nuclei (ONL, top) and RPE (bottom) layers are shown by the brackets. Images show a 45° field of view (∼1.4 mm scan length). (B) Representative images from each genotype at 11 months of age. (C,D) Quantification of RPE (C) and ONL (D) thickness of 11-month-old mice. Mean±s.e.m. Dots represent individual mice (the right and left eyes were averaged together). WT *n*=12; *Cp^−/−^ n*=7; *Cre^+^ Hepc^f/f^ n*=10; *Cre^+^ Hepc^f/f^ Cp^+/−^ n*=7; *Cre^+^ Hepc^f/f^ Cp^−/−^ n*=11. Statistical significance was assessed using Brown–Forsythe and Welch ANOVA test followed by Dunnett's T3 multiple comparisons test. Letters above groups designate significant differences: groups that have the same letter are not significantly different from each other.

Alongside the cSLO imaging, every month the mouse retinas were imaged *in vivo* using an optical coherence tomography (OCT) system. Rather than creating *en face* images like the cSLO, the OCT system takes cross-sectional images. These images showed that the *Cre^+^ Hepc^f/f^ Cp^−/−^* group developed RPE hypertrophy at about 8 months (Fig. 3A; note the brackets designating the RPE layer). This was followed by thinning of the outer nuclear layer (ONL) by 11 months (Fig. 3A, bottom; note the ONL brackets). The ONL is composed of photoreceptor nuclei and thins when photoreceptors die. Over the same timecourse, there were only very slight changes in the *Cre^+^ Hepc^f/f^* mice, primarily within the RPE layer (Fig. 3A, top). When comparing the genotypes, only the *Cre^+^ Hepc^f/f^ Cp^−/−^* and *Cre^+^ Hepc^f/f^ Cp^+/−^* groups exhibited RPE hypertrophy that was noticeable in the OCT images (Fig. 3B,C). On average, the RPE in the *Cre^+^ Hepc^f/f^ Cp^−/−^* and *Cre^+^ Hepc^f/f^ Cp^+/−^* groups were three times higher from the basal to apical side than the same region in WT RPE (Fig. 3C), although there was considerable variance among individual mice. There was also some slight RPE hypertrophy in the *Cre^+^ Hepc^f/f^* mice, with an average of 1.3-fold increase compared with the WT group (Fig. 3C). In the *Cp^−/−^* and *Cre^+^ Hepc^f/f^ Cp^−/−^* groups, the ONL thinned to about 75% of normal ONL thickness, whereas the rest of the groups remained statistically unchanged (Fig. 3D).

### mRNA analysis shows changes in levels of visual cycle, oxidative stress and macrophage transcripts

**Fig. 4.**
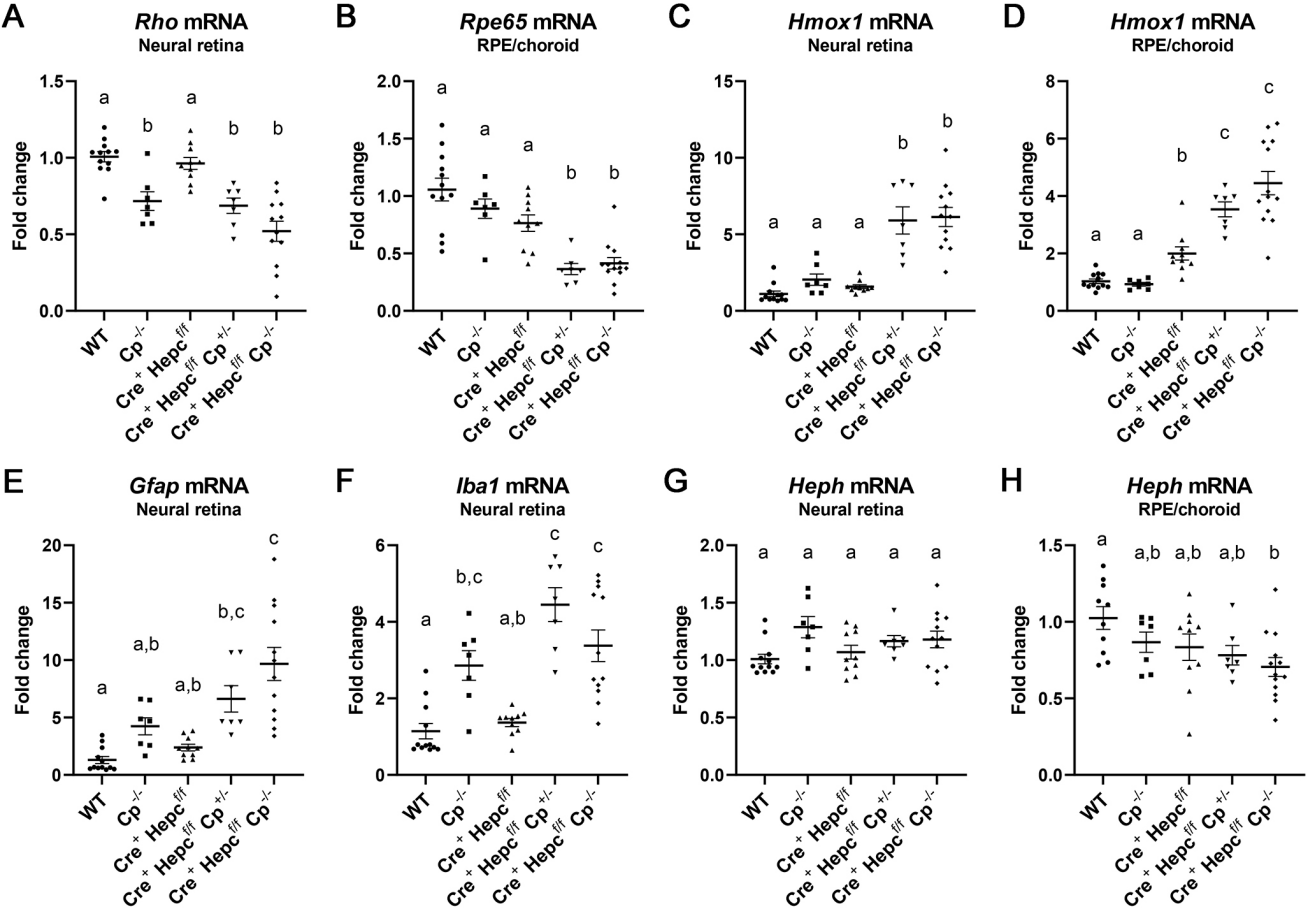
**qRT-PCR analysis of retina tissue.** (A-H) Fold change analysis of mRNA levels in neural retina (A,C,E-G) and RPE/choroid (B,D,H) of the following: (A) rhodopsin (*Rho*), (B) retinal pigment epithelium-specific 65 kDa protein (*Rpe65*), (C,D) heme oxygenase 1 (*Hmox1*), (E) glial fibrillary acidic protein (*Gfap*), (F) ionized calcium binding adaptor molecule 1 (*Iba1*) and (G,H) hephaestin (*Heph*). Mean±s.e.m. Dots represent individual mice. WT *n*=13 (except H *n*=11); *Cp^−/−^ n*=7; *Cre^+^ Hepc^f/f^ n*=10; *Cre^+^ Hepc^f/f^ Cp^+/−^ n*=7; *Cre^+^ Hepc^f/f^ Cp^−/−^ n*=12 (except B,D,H *n*=13). Statistical significance was assessed with a Brown–Forsythe and Welch ANOVA test followed by Dunnett's T3 multiple comparisons test. Letters above groups designate significant differences: groups that have the same letter are not significantly different from each other.

qRT-PCR was used to measure retinal and RPE health on a molecular level. Rhodopsin (*Rho*), which encodes a light-sensing photoreceptor-specific protein (Nathans, 1992), was used to measure general photoreceptor health (Fig. 4A). Whereas *Rho* mRNA decreased in the *Cp^−/−^*, *Cre^+^ Hepc^f/f^ Cp^+/−^* and *Cre^+^ Hepc^f/f^ Cp^−/−^* groups, it remained unchanged in the *Cre^+^ Hepc^f/f^* group (Fig. 4A). Retinal pigment epithelium-specific 65 kDa protein (*Rpe65*), which encodes an RPE-specific visual cycle protein (Wolf, 2005), was used as a marker of RPE health, and was decreased in the *Cre^+^ Hepc^f/f^ Cp^+/−^* and *Cre^+^ Hepc^f/f^ Cp^−/−^* groups (Fig. 4B). The antioxidant gene heme oxygenase 1 (*Hmox1*), used as a measure of oxidative stress (Keyse et al., 1990), increased significantly in the *Cre^+^ Hepc^f/f^ Cp^+/−^* and *Cre^+^ Hepc^f/f^ Cp^−/−^* groups in the neural retina and RPE/choroid (Fig. 4C,D). *Hmox1* levels were also increased in the *Cre^+^ Hepc^f/f^* group, but only in the RPE/choroid tissue (Fig. 4D). Glial fibrillary acidic protein (*Gfap*) mRNA increases when Müller cells are activated in response to retinal damage (Fernández-Sánchez et al., 2015). This increase was seen in the neural retina in the *Cre^+^ Hepc^f/f^ Cp^+/−^* groups and more prominently in the *Cre^+^ Hepc^f/f^ Cp^−/−^* group (Fig. 4E). Ionized calcium-binding adaptor molecule 1 (*Iba1*) mRNA increases when microglia and macrophages are activated (Ito et al., 1998), and a retinal increase was noted in the *Cp^−/−^*, *Cre^+^ Hepc^f/f^ Cp^+/−^* and *Cre^+^ Hepc^f/f^ Cp^−/−^* groups (Fig. 4F). Finally, there was no change in hephaestin (*Heph*), the membrane-bound analog of CP (Vulpe et al., 1999), with the exception of a decrease in the *Cre^+^ Hepc^f/f^ Cp^−/−^* group in the RPE/choroid (Fig. 4G,H). *Heph* mRNA levels were measured to ensure that *Heph* levels had not increased in response to the knocking out of *Cp*.

To summarize the qRT-PCR findings: (1) *Cre^+^ Hepc^f/f^* mice were rarely significantly different from WT mice in neural retina and RPE/choroid tissue at this time point of 11 months of age, (2) *Cp^−/−^* mice had decreased photoreceptor health and increased retinal microglial activation, but no change in oxidative stress levels, (3) *Cre^+^ Hepc^f/f^ Cp^−/−^* mice exhibited the most extreme changes in photoreceptor and RPE health (reduction) and oxidative stress (increase), and (4) *Cre^+^ Hepc^f/f^ Cp^+/−^* mice were more similar to the *Cre^+^ Hepc^f/f^ Cp^−/−^* mice than they were to any other group.

## DISCUSSION

Herein, we tested the hypothesis that when serum iron levels are elevated, CP plays a vital role in protecting the retina from oxidative stress. *Hepc* KO mice, a model of HH, experience elevated iron levels and age-dependent retinal autofluorescent lesions. These autofluorescent lesions are hypertrophic RPE cells and are eventually followed by photoreceptor degeneration. Knocking out *Cp* in these *Hepc* KO mice accelerated this process greatly, and at the 11-month time point, the severity of the damage in the *Hepc* KO mice harboring either heterozygous or homozygous *Cp* KO was significantly worse than in mice harboring the single KO of either *Hepc* or *Cp*. This was the case in terms of RPE damage (percentage blue autofluorescence on cSLO, RPE thickening on OCT, decreased *Rpe65* mRNA), Muller glial cell activation (increased *Gfap* mRNA), and increased neural retina and RPE oxidative stress (increased heme oxygenase mRNA). Within the *Cp/Hepc* DKO group, retinas from male mice were more damaged than those from females despite iron levels remaining mostly the same (Fig. S1 compares the sexes in WT and *Cp/Hepc* DKO mice; Figs S2-S5 break down the sexes for all genotypes). This may result from a retina-protective effect of estrogen, which has been suggested in the retina light-damage model (Wei et al., 2018).

This experiment adds to the growing body of evidence that ferrous iron is more toxic to the retina than ferric iron. For example, previously mice have been given an intravitreal injection of either ferrous sulfate or ferric sulfate (Shu et al., 2020). Although the retinas accumulated equal amounts of iron after both injections, only ferrous iron caused retinal degeneration. The ferric sulfate caused very little difference compared with the saline control in any of the assays used to test retinal health.

Unexpectedly, in this study *Cp* KO mice with WT *Hepc* had ONL thinning in the OCT images and corresponding decreased *Rho* mRNA levels, suggesting some retinal degeneration. These mice did have higher levels of iron in the liver, neural retina, and RPE than the WT controls, but not to the same extent as any of the mice that had *Hepc* knocked out. The cSLO images of the *Cp* KO mice did not show any autofluorescent lesions caused by RPE hypertrophy, but small autofluorescent puncta appeared, likely from microglial activation (images not shown). This was consistent with qRT-PCR analysis in the neural retina of the *Cp* KO mice showing elevated *Iba1*, which increases when microglia and macrophages are activated. Potentially, the increase of ferrous iron in the retina induced mild oxidative stress (as indicated by a small, but not significant, increase in *Hmox1* mRNA), which recruited microglia and/or blood-borne macrophages. These, in turn, may have been activated by elevated iron levels, leading them to kill the photoreceptors. There was no damage in the *Cp* KO mice noted in the RPE in any of the assays, which further differentiates the *Cp* KO mice from any groups that had *Hepc* knocked out.

The increased damage observed in the *Cp/Hepc* DKO mice compared with the *Hepc* KO mice can be primarily attributed to the increased proportion of iron existing in its more toxic ferrous state. Despite the iron levels increasing to similar levels, this ferrous iron would more readily undergo the Fenton reaction and form hydroxyl radicals, intensifying oxidative stress within the eye. Although the autofluorescent lesions developed at an accelerated rate in the *Cp/Hepc* DKO mice, the pattern of lesion formation, including their size and location, remained consistent between the two groups. Additionally, *Hepc* KO mice studied at a later time point displayed a similar RPE hypertrophy phenotype to the *Cp/Hepc* DKO group in this study, indicating a comparable mechanism of damage between the two groups (Baumann et al., 2019).

Alternatively, the observed differences in genotypes could be attributed to macrophage activation. Similar to the *Cp* KO group discussed earlier, but in contrast to the *Hepc* KO group, the *Cp/Hepc* DKO mice exhibited macrophage/microglia activation or recruitment (demonstrated by increased *Iba1* mRNA). This macrophage/microglia activation may have exacerbated the toxicity caused by the iron. Both the *Cp* and *Cp/Hepc* DKO groups had thinned ONLs, but there was no RPE hypertrophy in the *Cp* KO group, so the macrophage/microglia activation may have contributed to photoreceptor death in both models, but not the RPE hypertrophy observed in *Hepc* KO and *Cp/Hepc* DKO mice.

This study confirms that the combination of elevated iron levels and reduced CP levels contributed to bull's eye maculopathy in the HH patient mentioned in the introduction (Bellsmith et al., 2020). Of note, it did not matter if the *Hepc* KO mice were heterozygous or homozygous for the *Cp* knockout: both groups had about the same amount of pathology. This makes this issue more clinically relevant as people are more likely to have diminished CP levels than none at all. In particular, it has been shown that patients diagnosed with HH have significantly lower CP levels compared with controls (Cairo et al., 2001). Of the HH patients studied, more than a third of them had CP levels that were below the minimal normal value of 1.32 µmol/l. Retinal complications were unreported in the study, but, for context, the patient who developed the bull's eye maculopathy had lower CP levels (0.9 µmol/l) than almost all of the HH patients described by Cairo et al. (the lowest of which was also around 0.9 µmol/l) (Bellsmith et al., 2020; Cairo et al., 2001). Although the mechanism of correlation between HH and low CP levels was unknown, the lower CP levels observed in the HH patients were not attributed to copper deficiency, inflammatory conditions, liver damage, or elevated iron levels.

The development of HH seems to rely on unknown modulators on top of the known genetic factors. Meta-analyses have revealed that 80% of patients exhibit homozygosity for the C282Y mutation in the *HFE* gene (European Association for the Study of the Liver, 2010). However, among individuals homozygous for the C282Y mutation, only 14% of them actually develop HH. Hence, it is evident that additional factors are involved in the development of the disease. The findings from this study on *Cp/Hepc* DKO mice, along with the observation that patients diagnosed with HH have lower CP levels, suggests the significance of CP as one of these contributing factors. This emphasizes the need for close monitoring of HH patients for complications, such as retinal degeneration.

These findings also highlight the importance of monitoring CP levels in patients experiencing retinal degeneration. When CP levels are low, it is important to maintain normal iron levels to prevent further retinal complications caused by ferrous iron toxicity. Lowering iron levels is primarily done through phlebotomy, and iron chelation therapy serves as a viable secondary treatment option. Increasing CP levels could also be an important treatment strategy, although current methods for doing so are not well established. Systemic or intraocular gene therapy could be a promising avenue for further study for this purpose. Additionally, investigating the underlying causes of low CP levels, such as insufficient copper levels, is essential to uncover additional treatment possibilities.

## MATERIALS AND METHODS

### Generation of the mouse line

As previously described, a strain of C57Bl/6J mice was generated with hepcidin floxed (Lakhal-Littleton et al., 2016). These mice were then crossed with a strain that expresses Cre-recombinase under the control of the hepatocyte-specific promoter albumin (*AlbCre*; stock number 003574; The Jackson Laboratory) (Postic et al., 1999). These *AlbCre^+^ Hepc^f/f^* mice were then crossed with a strain of C57Bl/6J mice that had *Cp* knocked out (Harris et al., 1999) to generate a *Cp*/*Hepc* DKO mouse strain. The *Cp^−/−^* mice used in this study had floxed *Hepc* but no *AlbCre*. Some of the WT mice (*n*=5) had floxed *Hepc* without *AlbCre* and the rest had *Hepc* that was not floxed (*n*=8). The genotype of each mouse was verified when weaned and again when euthanized. The mice were free of the *rd1* and *rd8* mutations.

Both male and female mice were used. The two sexes were combined in the figures (refer to Figs S1-S5 for the sex differences). Mice were fed a standard laboratory diet *ad libitum* and maintained on a 12:12 h light/dark cycle. All procedures were approved by the Institutional Animal Care and Use Committee of the University of Pennsylvania (Philadelphia, PA, USA) and were performed in accordance with the Association for Research in Vision and Ophthalmology Statement for the Use of Animals in Ophthalmology and Vision Research.

### *In vivo* imaging and analysis

Both eyes of the mice were imaged every month using a Spectralis HRA cSLO (Heidelberg Engineering, Inc.) and Bioptigen Envisu R2200 ultra-high resolution (UHR) spectral-domain optical coherence tomography system (SD-OCT; Leica Microsystems).

Mice were prepared for *in vivo* imaging by applying one drop of a 2:1 mixture of 1% tropicamide and 2.5% phenylephrine (Akorn, Inc.) to both eyes several minutes prior to anesthesia. Anesthesia was achieved with an intraperitoneal injection of 93-98 mg/kg ketamine (Dechra Veterinary Products, Overland Park, KS, USA) and 10-11 mg/kg of xylazine (Akorn, Inc.). Soon thereafter, both corneas received topical anesthesia with 1% tetracaine (Alcon Laboratories, Inc.). Another few minutes later, Refresh Artificial Tears (Allergan, Irvine, CA, USA) was applied in conjunction with protective eye shields to prevent corneal desiccation and media opacity development (Bell et al., 2014). Mice were then moved to the cSLO imaging platform and imaged there before undergoing SD-OCT imaging subsequently.

cSLO images were collected using the blue autofluorescence (BAF) channel, which has a 486 nm raster scanned laser for excitation and 500-680 nm bandpass range for emission collection. Images were collected using the 105° Ultrawide Field (UWF) lens, which provides a ∼3.4 mm field of view (FOV) of the mouse fundus. System focus was trained on the RPE and images were collected with the optic nerve centrally located within the image FOV as previously described (Bell et al., 2015). Twenty-five frames were acquired in high-speed mode (768×768 pixels) from each mouse retina and automatically co-registered and averaged in real time using the Heidelberg Eye Explorer (HEYEX 1) software automatic real-time (ART) processing feature. Averaged images were auto-normalized for best contrast between hypo- and hyper-fluorescent features.

Following cSLO, SD-OCT images with a 45° FOV lens (∼1.4 mm scan length) were collected with the optic nerve centrally positioned. Orthogonal B-scans (1400 A-scans/2 B-scans×15 frames/B-scan) were collected at 0° and 90° to capture the horizontal and vertical meridians through the optic nerve. Upon completion of imaging, mice were administered 1.5 mg/kg of atipamezole HCL (Modern Veterinary Therapeutics, LLC., Miami, FL, USA) to induce recovery for anesthesia. Eyes were covered with Puralube Vet Ointment (Dechra Veterinary Products, Overland Park, KS, USA) to protect the cornea during recovery.

The 15 SD-OCT B-scan image frames from each orthogonal B-scan were co-registered and averaged using InVivoVue software v2.1 (Leica Microsystems). OCT images were analyzed using ImageJ (NIH, Bethesda, MD, USA). The upper and lower boundaries of the RPE and ONL were outlined and a custom macro was used to measure the average distance between the outlined boundaries. Because of the high inter-eye correlation between the left and right eyes, the average thickness of the horizontal and vertical OCT images of both eyes were reported.

For cSLO images, two researchers who were unaware of the experimental groupings manually quantified the amount of blue (488 nm) autofluorescence. To accomplish this in ImageJ, the threshold tool was adjusted to include only the bright pixels that were part of the hyper-autofluorescent lesions. ImageJ reported the percentage of the retina above the threshold, which was then recorded. The measurements from each researcher were averaged, and, because of the high inter-eye correlation, the measurements from the right and left eyes were then averaged.

### Collection of tissues

Mice were euthanized at 11 months of age by an intraperitoneal injection of 93-98 mg/kg ketamine (Dechra Veterinary Products, Overland Park, KS, USA) and 10-11 mg/kg of xylazine (Akorn, Inc.), followed by cervical dislocation. The right eyes were enucleated, placed in cold PBS (Thermo Fisher Scientific), and the cornea, iris, lens and neural retina were dissected out. The neural retina and RPE/choroid were flash-frozen on dry ice for qRT-PCR analysis. The left eyes were enucleated and fixed in 4% paraformaldehyde (Electron Microscopy Science) for 15 min, followed by the removal of the cornea, iris and lens. These eye cups were then put in a 30% sucrose (Thermo Fisher Scientific) solution for 24 h to be subsequently cryosectioned for epifluorescence microscopy. Sera was harvested by keeping collected blood at room temperature for 30 min to allow it to clot, and then was centrifuged for 30 min at 3000 rpm (830 ***g***). The sera was then removed by pipette and stored at −20°C until subsequent analysis of iron levels. Livers were dissected out, flash-frozen and stored at −80°C for iron quantification. Tail samples were collected and submitted to Transnetyx (Memphis, TN, USA) for genotyping.

### qRT-PCR

RNA was isolated from either homogenized neural retina or RPE/choroid samples using the QIAGEN RNeasy kit, following the manufacturer's protocol. The RNA yield was quantified using a NanoDrop spectrophotometer and was then reverse transcribed into cDNA using TaqMan Reverse Transcription Reagents (Applied Biosystems), according to the manufacturer's protocol. qRT-PCR was performed on a 7500 Fast Real-Time PCR System (Applied Biosystems) using the following TaqMan probes (Applied Biosystems): *Gapdh* (Mm99999915_g1), *Gfap* (Mm01253033_m1), *Heph* (Mm01224864_m1), *Hmox1* (Mm00516005_m1), *Iba1* (Mm00479862_g1), *Rho* (Mm00520345_m1), *Rpe65* (Mm00504133_m1) and *Tfrc* (Mm00441941_m1). All reactions were performed in technical triplicate and analyzed using the comparative CT method (Schmittgen and Livak, 2008): ΔCT values were obtained by normalizing the CT values to their respective *Gapdh* CT values, and then the ΔΔCT values were obtained by normalizing the ΔCT value of each sample to the averaged ΔCT value of all WT mice for each probe. The fold change was calculated by taking 2 to the negative power of the ΔΔCT value.

### Epifluorescence microscopy

After the eyes were fixed, dissected and dehydrated as described above, they were embedded in Tissue-Tek O.C.T. Compound (Sakura Finetek USA, Inc.). They were then flash-frozen and stored at −20°C. The samples were cut into 10 µm sections using a Leica CM3050 S cryostat (Leica Biosystems). No bleaching was done on the samples. The nuclei were stained with DAPI Fluoromount-G (SouthernBiotech) and the sections were imaged by epifluorescence microscopy at identical exposure parameters with the Nikon Eclipse 80i microscope and DS-QiMc camera (Nikon), using Nikon Elements software.

### Iron quantification assays

Serum iron levels were quantified using the MAK025 iron assay kit (Sigma-Aldrich), following the manufacturer's protocol.

To quantify liver iron levels, the livers were first digested at 65°C in an acid digest solution [3 M HCl (Sigma-Aldrich), 10% trichloroacetic acid (Sigma-Aldrich)] overnight, with 1 ml of acid digest for every 100 mg of tissue. After digestion, the samples were vortexed and centrifuged at 830 ***g*** for 5 min, and then 20 µl of the supernatant was added to 1 ml of chromogen reagent [2.25 M sodium acetate (Sigma-Aldrich) pretreated with Chelex 100 (Bio-Rad), 0.01% bathophenanthroline (Sigma-Aldrich), 0.1% thioglycolic acid (Sigma-Aldrich)]. The absorbances were read at 535 nm using a spectrometer (Beckman DU-640, Beckman Coulter) and the iron levels were calculated by comparing the absorbances with a serial dilution of iron standard (Sigma-Aldrich) mixed with the chromogen reagent.

### Statistical analysis

Because the effect size was unknown, sample size was determined by the maximum number of mice available. One WT mouse was excluded because of an unknown immune response causing microglial or macrophage activation that led to retinal degeneration. The exclusion was decided after the study was complete. Mice were identified by their ID number only and researchers for outcome assessment were unaware of their genotype.

Statistical analysis was carried out in Prism 9.2 (GraphPad Software). The Brown–Forsythe and Welch one-way ANOVA was used because the variances were not equal across comparison groups. Post-hoc pairwise comparisons using Dunnett's T3 multiple comparison test were used because of small sample size (*n*<50) (Dunnett, 1980). When analyzing sex and genotype concurrently (Fig. S1), a standard two-way ANOVA was used followed by post-hoc pairwise comparisons using Holm– Šidák's multiple comparisons test.

## Supplementary Material

10.1242/dmm.050226_sup1 Supplementary information
