# Supplementary material for: Low ceruloplasmin levels exacerbate retinal degeneration in a hereditary hemochromatosis model
Source: Dis Model Mech. 2023 Jul 13;16(7):dmm050226. doi: 10.1242/dmm.050226 (PMC10354715; doi:10.1242/dmm.050226)
Supplement: Supplementary information [file dmm-16-050226-s1.pdf]

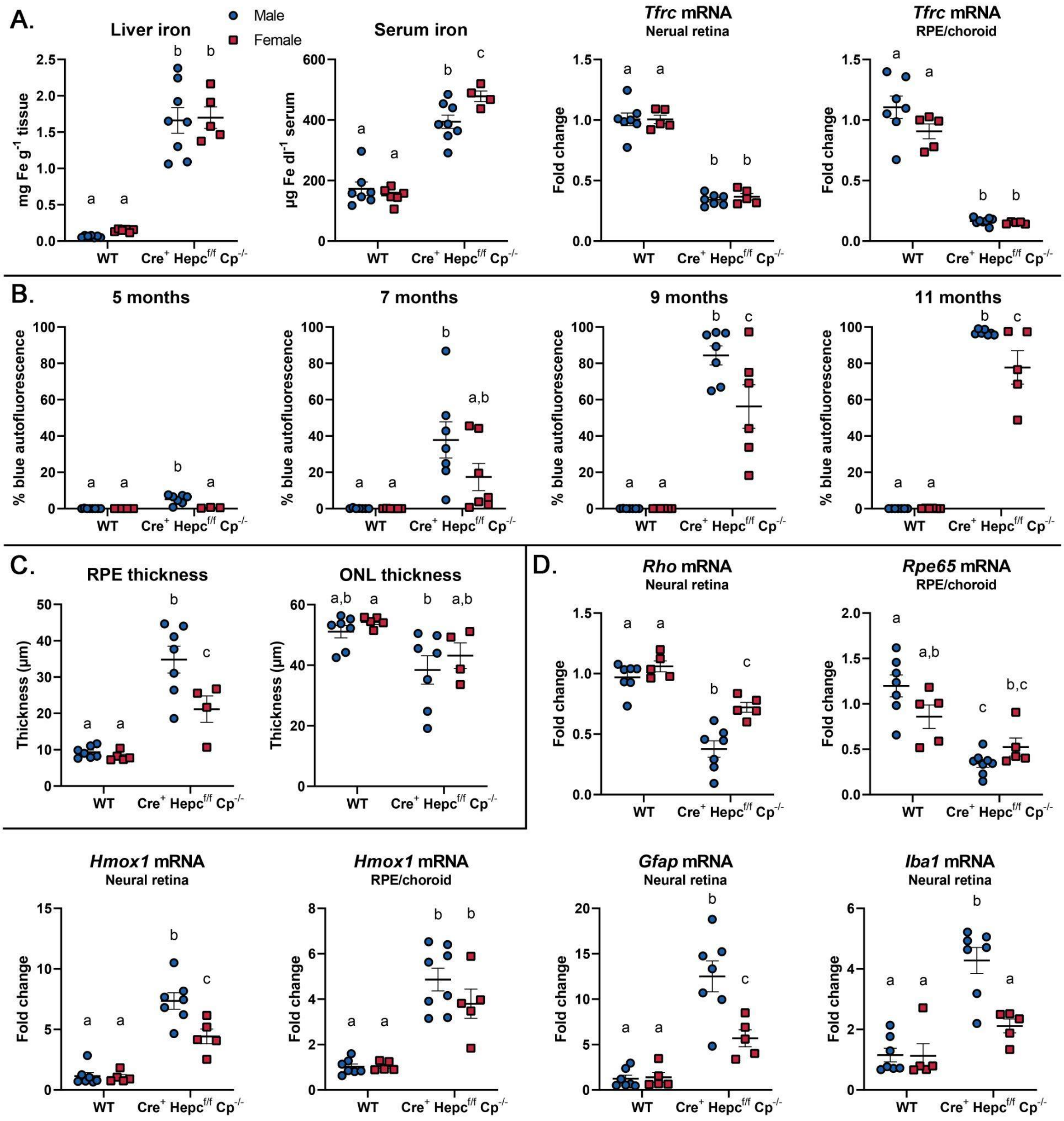

Disease Models & Mechanisms • Supplementary information

Fig. S1. Comparison of male and female WT and *Cp/Hepc* DKO mice, corresponding to figures 1-4. (A) Iron analysis in the liver, serum, neural retina, and RPE/choroid. **(B)** Percentage of cSLO image area containing blue autofluorescence at 5, 7, 9, and 11 months of age. **(C)** Thickness of RPE and outer nuclear layers (ONL) as seen at 11 months of age in OCT images. **(D)** qRT-PCR analysis of neural retina and RPE/choroid tissue using rhodopsin (*Rho*), retinal pigment epithelium-specific 65 kDa protein (*Rpe65*), heme oxygenase 1 (*Hmox1*), glial fibrillary acidic protein (*Gfap*), and ionized calcium binding adaptor molecule 1 (*Iba1*). This figure separates the male and female data in WT and *Cp/Hepc* DKO mice within the data presented in **(A)** figure 1, **(B)** figure 2, **(C)** figure 3, and **(D)** figure 4. Mean ± s.e.m. is reported with dots representing individual mice. Statistics were done using a 2-way ANOVA followed by Holm-Sidak's multiple comparisons test. Letters above groups designate significant differences: groups that have the same letter are not significantly different from each other. The rest of the genotypes are reported in supplemental figures 2-5.

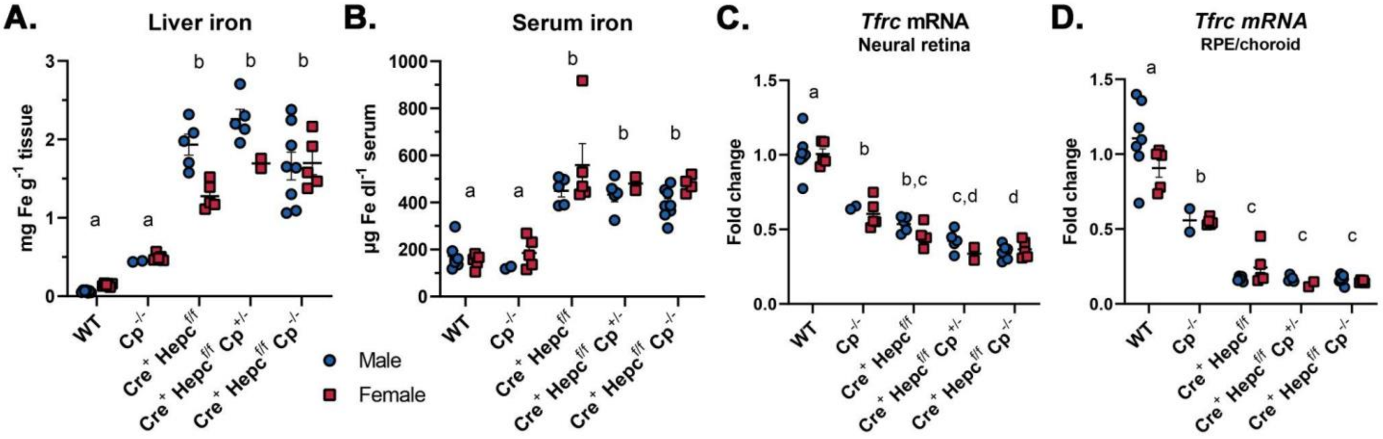

Fig. S2. Comparison of iron levels in male and female mice in liver, serum, and retinal tissue, corresponding to figure 1. (A) Liver iron levels in male (blue) and female (red) mice, measured in mg iron per g of liver. (B) Serum iron levels measured in µg per dl. (C) Neural retinal and (D) RPE/choroid *Tfrc* mRNA levels as an indicator of intracellular iron levels; as iron increases *Tfrc* mRNA is degraded. Bars represent mean ± s.e.m. Dots represent individual mice with the following distribution: WT male n=7, female n=6; $Cp^{-/-}$ male n=2, female n=5; Cre^{+} $Hepc^{f/f}$ male n=5, female n=5; Cre^{+} $Hepc^{f/f}$ $Cp^{+/-}$ male n=5, female n=2; Cre^{+} $Hepc^{f/f}$ $Cp^{-/-}$ male n=8 (except Fig. 1C n=7), female n=5. Statistics were done comparing genotypes (with the sexes combined due to a low n for some groups) with a Brown-Forsythe and Welch ANOVA test followed by Dunnett's T3 multiple comparisons test. Letters above groups designate significant differences: genotypes that have the same letter are not significantly different from each other.

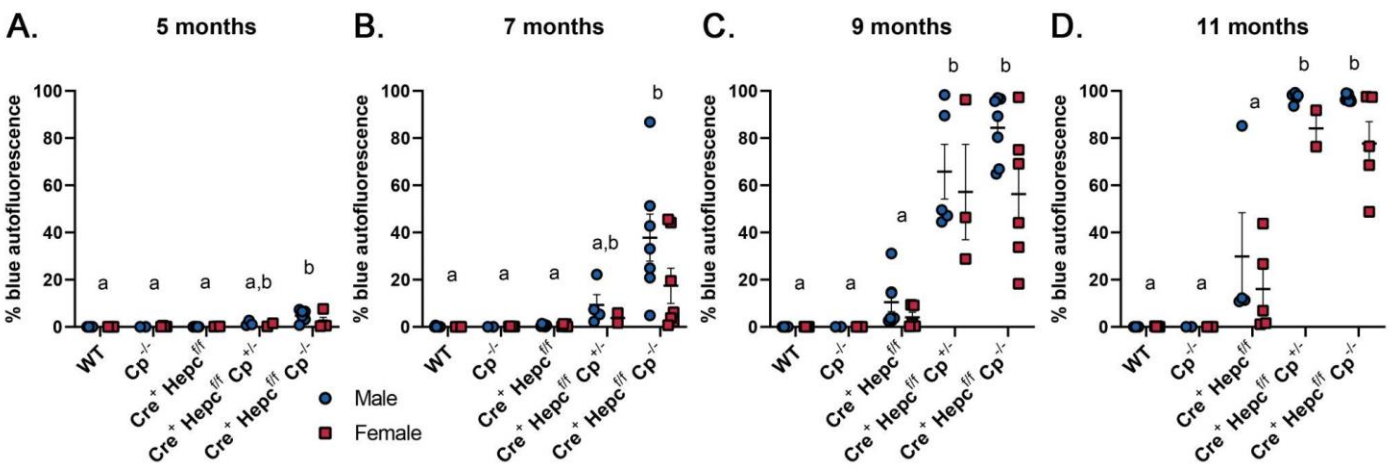

Fig. S3. Blue autofluorescence measured in cSLO images in male and female mice, corresponding to figure 2. (A) Percentage of image area with blue autofluorescence in each cSLO image in male (blue) and female (red) mice at 5 months, **(B)** 7 months, **(C)** 9 months, and **(D)** 11 months. Mean ± s.e.m is reported. Dots represent individual mice with the following distribution: 5 mo. WT male n=7 female n=5, *Cp*$^{-/-}$ male n=2 female n=4, *Cre*$^{+}$ *Hepc*$^{f/f}$ male n=7 female n=3, *Cre*$^{+}$ *Hepc*$^{f/f}$ *Cp*$^{+/-}$ male n=3 female n=2, *Cre*$^{+}$ *Hepc*$^{f/f}$ *Cp*$^{-/-}$ male n=6 female n=4; 7 mo. WT male n=7 female n=6, *Cp*$^{-/-}$ male n=2 female n=5, *Cre*$^{+}$ *Hepc*$^{f/f}$ male n=6 female n=5, *Cre*$^{+}$ *Hepc*$^{f/f}$ *Cp*$^{+/-}$ male n=4 female n=2, *Cre*$^{+}$ *Hepc*$^{f/f}$ *Cp*$^{-/-}$ male n=7 female n=7; 9 mo. WT male n=7 female n=6, *Cp*$^{-/-}$ male n=2 female n=5, *Cre*$^{+}$ *Hepc*$^{f/f}$ male n=7 female n=5, *Cre*$^{+}$ *Hepc*$^{f/f}$ *Cp*$^{+/-}$ male n=5 female n=3, *Cre*$^{+}$ *Hepc*$^{f/f}$ *Cp*$^{-/-}$ male n=7 female n=6; 11 mo. WT male n=7 female n=6, *Cp*$^{-/-}$ male n=2 female n=5, *Cre*$^{+}$ *Hepc*$^{f/f}$ male n=4 female n=5, *Cre*$^{+}$ *Hepc*$^{f/f}$ *Cp*$^{+/-}$ male n=5 female n=2, *Cre*$^{+}$ *Hepc*$^{f/f}$ *Cp*$^{-/-}$ male n=7 female n=5. Statistics were done comparing genotypes (with the sexes combined due to a low n for some groups) with a Brown-Forsythe and Welch ANOVA test followed by Dunnett's T3 multiple comparisons test. Letters above groups designate significant differences: genotypes that have the same letter are not significantly different from each other.

Disease Models & Mechanisms • Supplementary information

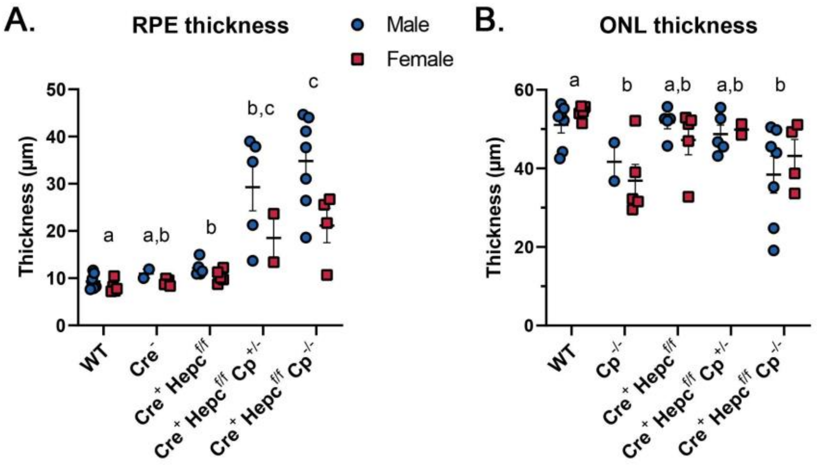

Fig. S4. Thickness of RPE and ONL in OCT images for male and female mice, corresponding to figure 3. (A) Quantification of RPE and **(B)** ONL thickness of male (blue) and female (red) 11-month-old mice. Mean ± s.e.m. is reported. Dots represent individual mice with the following distribution: WT male n=7, female n=5; *Cp*$^{-/-}$ male n=2, female n=5; *Cre*$^{+}$ *Hepc*$^{f/f}$ male n=5, female n=5; *Cre*$^{+}$ *Hepc*$^{f/f}$ *Cp*$^{+/-}$ male n=5, female n=2; *Cre*$^{+}$ *Hepc*$^{f/f}$ *Cp*$^{-/-}$ male n=7, female n=4. Statistics were done comparing genotypes (with the sexes combined due to a low n for some groups) with a Brown-Forsythe and Welch ANOVA test followed by Dunnett's T3 multiple comparisons test. Letters above groups designate significant differences: genotypes that have the same letter are not significantly different from each other.

Disease Models & Mechanisms • Supplementary information

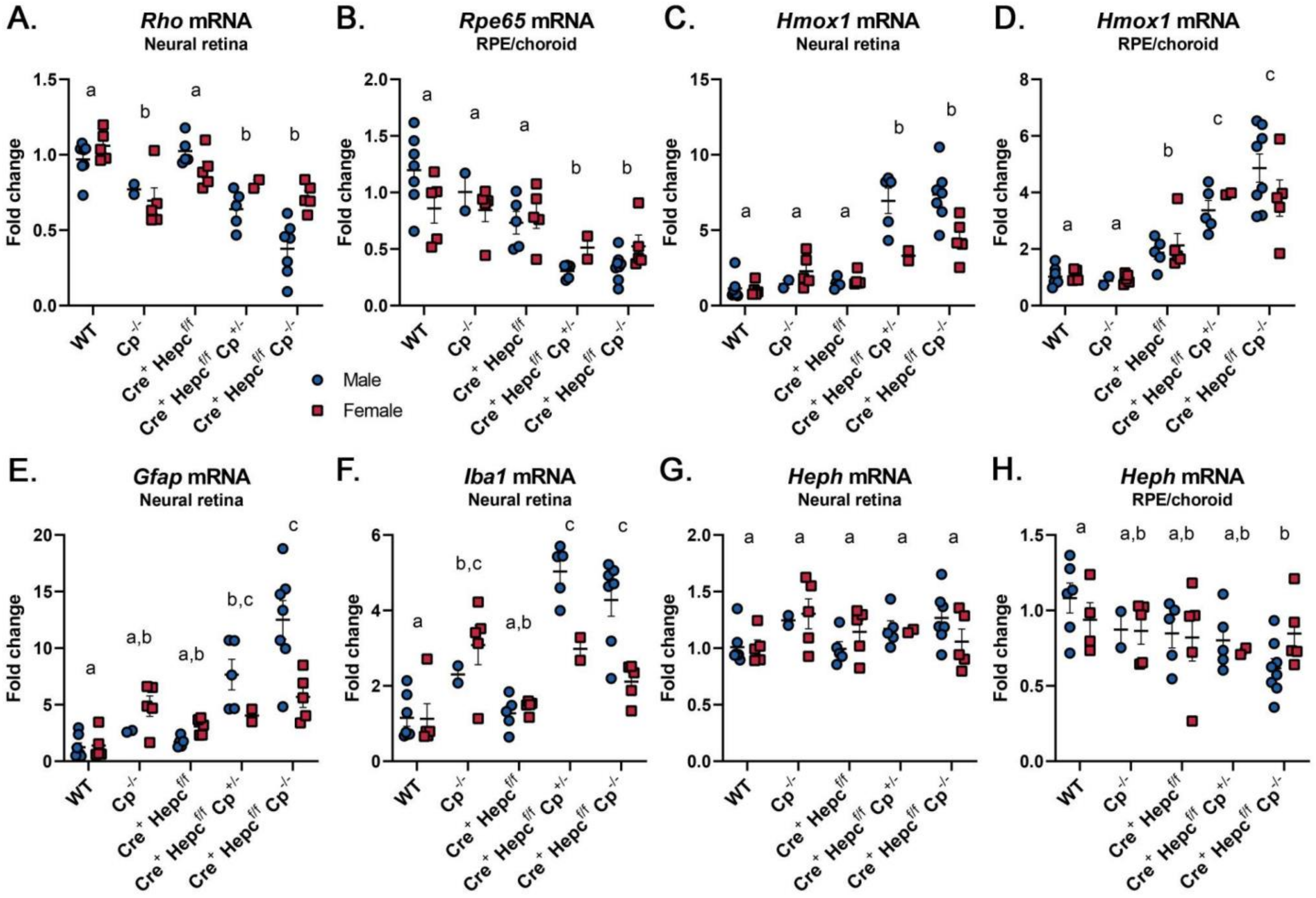

Fig. S5. qRT-PCR results for neural retinal and RPE tissue in male and female mice, corresponding to figure 4. Fold change analysis of mRNA levels in **(A, C, E, F, G)** neural retina and **(B, D, H)** RPE/choroid of male (blue) and female (red) mice. Analysis was done using the following probes: **(A)** rhodopsin (*Rho*), **(B)** retinal pigment epithelium-specific 65 kDa protein (*Rpe65*), **(C-D)** heme oxygenase 1 (*Hmox1*), **(E)** glial fibrillary acidic protein (*Gfap*), **(F)** ionized calcium binding adaptor molecule 1 (*Iba1*), and **(G-H)** hephaestin (*Heph*). Mean ± s.e.m. is reported. Dots represent individual mice with the following distribution: WT male n=7 (except Fig. H n=6), female n=5 (except Fig. H RPE n=4); $Cp^{-/-}$ male n=2, female n=5; Cre^{+} $Hepc^{f/f}$ male n=5, female n=5; Cre^{+} $Hepc^{f/f}$ $Cp^{+/-}$ male n=5, female n=2; Cre^{+} $Hepc^{f/f}$ $Cp^{-/-}$ male n=7 (Fig. B,D,H n=8), female n=5. Statistics were done comparing genotypes (with the sexes combined due to a low n for some groups) with a Brown-Forsythe and Welch ANOVA test followed by Dunnett's T3 multiple comparisons test. Letters above groups designate significant differences: genotypes that have the same letter are not significantly different from each other.

Disease Models & Mechanisms • Supplementary information
